# Influence of surface chemical properties on the toxicity of engineered zinc oxide nanoparticles to embryonic zebrafish

**DOI:** 10.3762/bjnano.6.160

**Published:** 2015-07-20

**Authors:** Zitao Zhou, Jino Son, Bryan Harper, Zheng Zhou, Stacey Harper

**Affiliations:** 1 School of Chemical, Biological and Environmental Engineering, Oregon State University, Corvallis, Oregon, 97330, United States; 2 Department of Environmental and Molecular Toxicology, Oregon State University, Corvallis, Oregon, 97330, United States; 3 Oregon Nanoscience and Microtechnologies Institute, Eugene, Oregon, United States

**Keywords:** kriging estimation, modelling, nanomaterials, nanotechnology, toxicology

## Abstract

Zinc oxide nanoparticles (ZnO NPs) are widely used in a variety of products, thus understanding their health and environmental impacts is necessary to appropriately manage their risks. To keep pace with the rapid increase in products utilizing engineered ZnO NPs, rapid in silico toxicity test methods based on knowledge of comprehensive in vivo and in vitro toxic responses are beneficial in determining potential nanoparticle impacts. To achieve or enhance their desired function, chemical modifications are often performed on the NPs surface; however, the roles of these alterations play in determining the toxicity of ZnO NPs are still not well understood. As such, we investigated the toxicity of 17 diverse ZnO NPs varying in both size and surface chemistry to developing zebrafish (exposure concentrations ranging from 0.016 to 250 mg/L). Despite assessing a suite of 19 different developmental, behavioural and morphological endpoints in addition to mortality in this study, mortality was the most common endpoint observed for all of the ZnO NP types tested. ZnO NPs with surface chemical modification, regardless of the type, resulted in mortality at 24 hours post-fertilization (hpf) while uncoated particles did not induce significant mortality until 120 hpf. Using eight intrinsic chemical properties that relate to the outermost surface chemistry of the engineered ZnO nanoparticles, the highly dimensional toxicity data were converted to a 2-dimensional data set through principal component analysis (PCA). Euclidean distance was used to partition different NPs into several groups based on converted data (score) which were directly related to changes in the outermost surface chemistry. Kriging estimations were then used to develop a contour map based on mortality data as a response. This study illustrates how the intrinsic properties of NPs, including surface chemical modifications and capping agents, are useful to separate and identify ZnO NP toxicity to zebrafish (*Danio rerio*).

## Introduction

Accelerated advancements in nanotechnology and nanoscience have found applications in a variety of scientific fields, leading to a rapid increase in the types of engineered nanoparticles on the market. In particular, zinc oxide nanoparticles (ZnO NPs) are the third highest production volume nanoparticles at roughly 550 tons per year [1]. Given their value as UV-protects [2], self-cleaning surfaces [3], sensors [4] and catalysts [5], it is expected that the use of engineered ZnO NPs will continue to increase with the increasing market demand. Such widespread use will also inevitably result in increased environmental release and a higher potential for human exposure [6]. As such, understanding which features of ZnO NPs increase their risks to humans and/or the environment is of paramount importance [7]. Despite this fact, very few studies to date have looked across a wide-range of engineered ZnO nanoparticle types to investigate how surface chemical modifications alter toxicity.

The toxicity of ZnO NPs to a wide range of species can be found elsewhere in literature from in vivo [8–9] to in vitro studies [10–11]. Bare ZnO NPs (lacking surface ligands) are known to cause delayed embryo hatching, developmental abnormalities [12] through dissolution and release of ionic zinc [13–14] as well as induction of DNA damage through generation of reactive oxidative species (ROS) [12,15]. ZnO NPs are often coated with a variety of capping agents or surface ligands with differing chemical properties to functionalize the surface and improve stability against agglomeration and dispersibility in a given medium [16]. These surface alterations have the potential to alter their toxicity as a result of differences in the release of Zn^2+^ ions and ROS production compared to bare ZnO NPs [17–18]. In addition, the behaviour of surface functionalized ZnO NPs may vary compared to non-functionalized (bare) ZnO NPs by altering stability and/or agglomeration, potentially altering bioavailability and toxicity to aquatic organisms [18–21]. While the dissolution kinetics and agglomeration state of the ZnO NPs is known to influence the toxicity of the materials, this study aimed to determine if specific intrinsic features could be used in lieu of empirical data on the material’s behaviour.

Surface chemical ligands and capping agents are more closely related to the fate and effects of ZnO NPs than the core composition alone [18–19,22]. Thus, it is expected that surface chemical properties can be employed as descriptors to model the toxicity of various types of engineered ZnO NPs. The development of such relationships between a set of intrinsic properties of ligands and/or capping agents with their biological effects could serve as the basis of nanomaterial structure–activity relationships (nanoSARs) [23–24]. However, there is a limited understanding of how to link different nanoparticle surface chemistries directly to the fate and effects of ZnO NPs in organisms, and whether these properties can be used to develop predictive models useful in the development of safer engineered ZnO materials [7].

**Figure 1:**
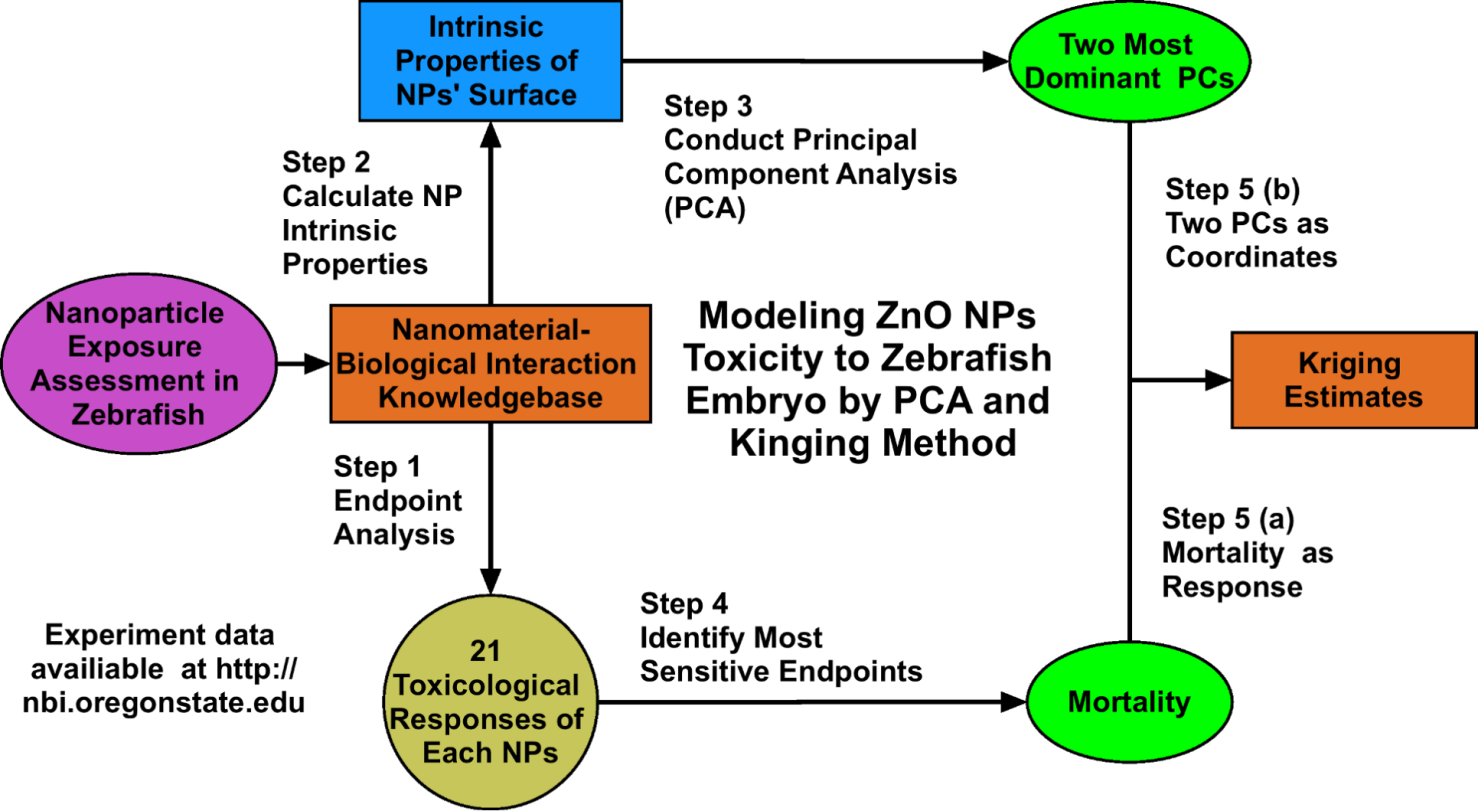
Data processing for model development.

The main objective of this study were 1) to investigate whether the intrinsic properties of different capping agents or surface ligands of engineered ZnO NPs alter their toxicity and 2) to determine if these features can be used to model the developmental toxicity of ZnO nanoparticles to embryonic zebrafish (*Danio rerio*) (Figure 1). Zebrafish embryos were selected as vertebrate test species as their transparent tissues allow for easy visual assessment of multiple developmental malformations and their rapid development makes them ideal for studies of numerous types of NPs [25–26]. Due to the agglomeration of ZnO NPs in fishwater, the chorionic membrane can serve as a barrier to the direct interaction of NPs or dissolved oxygen with the developing embryo, thus we chose to remove this barrier in our study. The removal also allows for the visual analysis of the developing embryo, which can be hampered when the chorion is intact and coated with nanoparticles [25,27]. To achieve these objectives, we conducted zebrafish embryo toxicity testing for 17 different types and sizes of ZnO NPs with differing surface chemistries. Then, using bare and surface modified NP toxicity data and eight intrinsic chemical properties related to the outermost surface chemistry, we conducted principal component analysis (PCA) to extract descriptors useful as coordinates to develop a model of how surface chemistry impacts ZnO NP toxicity.

Selected surface features used in the PCA were those deemed likely to influence biological interactions with the NP surface. Size (SZ) was chosen as it has been reported by others to influence NP toxicity [11,28]. Hydrophobicity was selected as the Log P (partitioning coefficient) of NPs has been found to be related to toxic responses in other organisms [29]; however, since ZnO NPs can release zinc ions [30] and Log P is pH-independent [31], distribution coefficient (Log D) was also considered for both ionic and non-ionic forms. Polarizability was selected (PL) as a factor to describe the molecules electronic properties and its ability to change with external fields in biochemical reactions [32]. Polar surface area (PS) represents the area formed by the polar areas of the molecule and has been used to predict drug intestinal absorption in humans, thus it may be a useful predictor of other biological interactions [33]. Van der Waals (VDW) surface area calculated by VDW radius, is associated with the likelihood of NP agglomeration [34]. Solvent accessible surface area (SASA) can be used to estimate the protein-ligand binding free energy [35], and molar refractivity (RF) represents the energy required to polarize one mole of the substance and is associated with receptor binding affinity [36]. Dreiding energy (DE) will be used to predict the binding affinity of organic molecules with Zn and membrane proteins [37]. Although zeta potential is known to be crucial to biological response [38]; it’s dependent on the environment in which it is measured and thus is not an intrinsic feature of the NP and thus was omitted from the model.

Following PCA, the ordinary kriging (OK) method was applied to estimate the pattern of variation of mortality in a given coordinate system. We hypothesized that surface chemical modifications would result in significant alterations in toxicity that would depend on the type of surface chemical modification performed.

## Results

### Estimation of intrinsic capping agent properties

**Figure 2:**
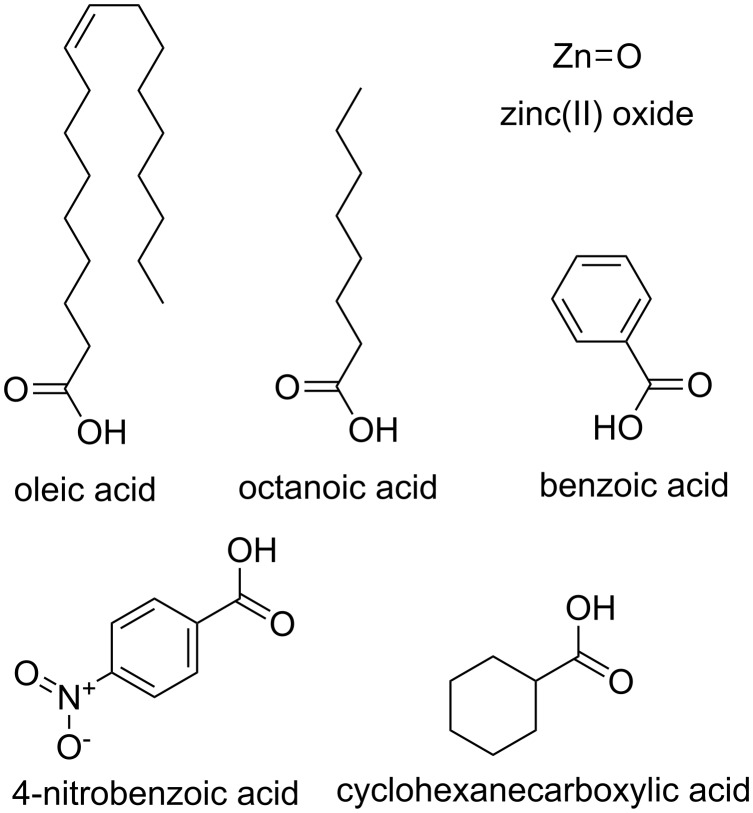
Chemical structures used to calculate the surface properties.

The 17 ZnO NPs (Table 1) had 6 different surface chemistries including bare ZnO, oleic acid, octanoic acid, para-nitrobenzoic acid, cyclohexanecaboxylic acid and benzoic acid (Figure 2). The average primary particle sizes in this study ranged from 4 to 70 nm (Table 1). Table 2 provides the values calculated for the intrinsic features of the 6 surface chemistries. The calculated distribution coefficient (Log D) had the least variance of all the parameters ranging from −1.22 to 5.62. Van der Waal surface area is the surface of the union of the spherical atomic surfaces defined by the van der Waals radius of each component atom in the molecule. Van der Waal surface area values for bare ZnO were 50.3 Å^2^ and ranged from 173 to 560.40 Å^2^ for other surface chemistries. These values had the highest variance in our estimations.

**Table 1:** Description of zinc oxide nanoparticles included in this study (17 in total).

NBI record	Particle descriptor	Manufacturer	Surface group	Size (nm)

nbi_085	ZnO + oleic acid	Voxtel	oleic acid	62
nbi_086	ZnO + oleic acid	Voxtel	oleic acid	26
nbi_087	ZnO	Sigma-Aldrich	—	62
nbi_088	ZnO	Voxtel	—	26
nbi_089	ZnO + octanoic acid	Voxtel	octanoic acid	62
nbi_090	ZnO + octanoic acid	Voxtel	octanoic acid	26
nbi_091	ZnO + para-nitrobenzoic acid	Voxtel	para-nitrobenzoic acid	62
nbi_092	ZnO + para-nitrobenzoic acid	Voxtel	para-nitrobenzoic acid	26
nbi_093	ZnO + cyclohexane carboxilic acid	Voxtel	cyclohexane carboxilic acid	62
nbi_094	ZnO + cyclohexane carboxilic acid	Voxtel	cyclohexane carboxilic acid	26
nbi_095	ZnO + benzoic acid	Voxtel	benzoic acid	62
nbi_096	ZnO + benzoic acid	Voxtel	benzoic acid	26
nbi_136	ZnO	Boise State University	—	14.6
nbi_137	ZnO	Boise State University	—	33.6
nbi_138	ZnO	Boise State University	—	4.5
nbi_139	ZnO	Boise State University	—	10.2
nbi_187	NanoGard ZnO (NGZ)	Alfa Aesar, NanoGard, Prod.#44898, lot#D28X017	—	70

**Table 2:** Intrinsic properties of different surface chemistries.

Intrinsic descriptor	Oleic acid	Octanoic acid	4-Nitrobenzoic acid	Cyclohexane carboxylic acid	Benzoic acid	Zinc oxide

Log D	5.62	0.53	−1.22	−0.43	−1.08	−0.20
Polarizability (Å^3^)	34.5	16.1	15.8	13.4	13.2	1.00
Polar surface area (Å^2^)	37.3	37.3	83.1	37.3	37.3	17.1
VDW surface area (Å^2^)	560	283	211	221	173	50.3
Solvent-accessible surface area (Å^2^)	689	403	330	260	284	156
Molar refractivity (cm^3^/mol)	87.1	40.7	39.7	39.7	33.2	1.44
Dreiding energy (kcal/mol)	35.7	12.1	23.1	24.8	16.6	0.00

### ZnO nanoparticle toxicity

**Figure 3:**
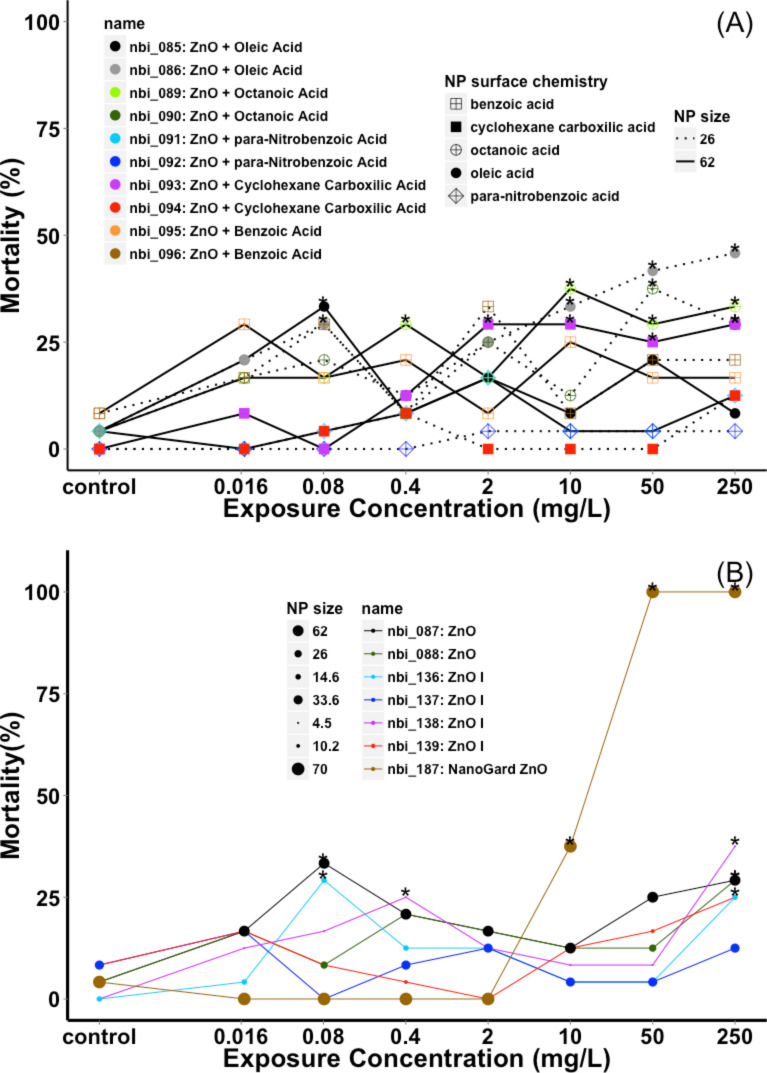
Zebrafish mortality at 120 hpf following exposure to: (A) ZnO NPs with and (B) without surface modification.

Embryonic zebrafish mortality was concentration dependent and varied with different types of bare and surface engineered ZnO NPs as expected. Mortality for the bare and surface modified ZnO NPs as a function of exposure concentration is shown in Figure 3. Surface modified ZnO particles caused significant mortality at 24 hpf, in some cases at exposure concentrations as low as 0.08 mg/L; however, despite the exposures continuing until 120 hpf, no significant mortality or developmental problems were noted after 24 hpf (Figure 3A). Bare ZnO NPs showed similar results with 2 out of 7 displaying no visible signs of toxicity at the highest concentration tested (Figure 3B). In contrast to the surface engineered particles, the toxicity of bare particles occurred more frequently at 120 hpf (3 out of 7 materials, Supporting Information File 1). Bare NanoGard ZnO (NGZ) showed the highest 120 hpf mortality of all the tested particles (bare and surface modified) with 100% mortality (*n* = 24 embryos) at 50 mg/L. In addition, NGZ was the only ZnO particle tested (bare or surface modified) that resulted in any significant sublethal responses, eliciting swim bladder malformations at 10 mg/L and notochord malformations at the highest exposure concentration (see Supporting Information File 2). The results of the endpoint analysis using the Fisher’s exact test for all tested NPs are provided in Supporting Information File 1. Detailed raw toxicity data for each individual exposure is also available online from the Nanomaterial-Biological Interactions knowledgebase (nbi.oregonstate.edu) [39].

Analysis of the 5 pairs of surface modified particles, with the same surface chemistries and differing average particle sizes, showed no clear trend related to the primary particle size (Figure 3A). Smaller oleic acid coated ZnO NPs (26 nm) caused significant mortality at the highest test concentration that did not occur for the larger (62 nm) oleic acid functionalized particles. In contrast, the larger octanoic acid coated ZnO NPs caused significant mortality at 0.4 mg/L while the smaller 26 nm particles did not induce toxicity until exposure concentrations reached 50 mg/L. Similarly, the ZnO NPs coated with cyclohexane carboxylic acid had a significantly different mortality rate between sizes, with the larger particles being more toxic than the smaller version (*p* = 0.009, 0.234 respectively).

### Principal components analysis

By selecting the most dominant components to explain the majority of data variance, PCA effectively reduced the dimensions of the dataset with keeping most information. It eliminated the correlation between different independent variables by creating different linear combinations which are independent of each other [40]. PCA was conducted on the database that consists of 8 property descriptors: size (SZ), Log D, polarizability (PL), polar surface area (PS), van der Waals surface (VS), solvent-accessible surface area (SASA), molar refractivity (RF) and Dreiding energy (DE) with 10 surface modified and 7 bare ZnO NPs (17 ZnO NP datasets × 8 properties). Each individual NP exposure dataset is comprised of results from experiments conducted at 8 exposure concentrations, thus the final matrix of the database was comprised of 136 rows and 8 columns (17 materials × 8 concentrations × 8 surface chemical properties).

**Figure 4:**
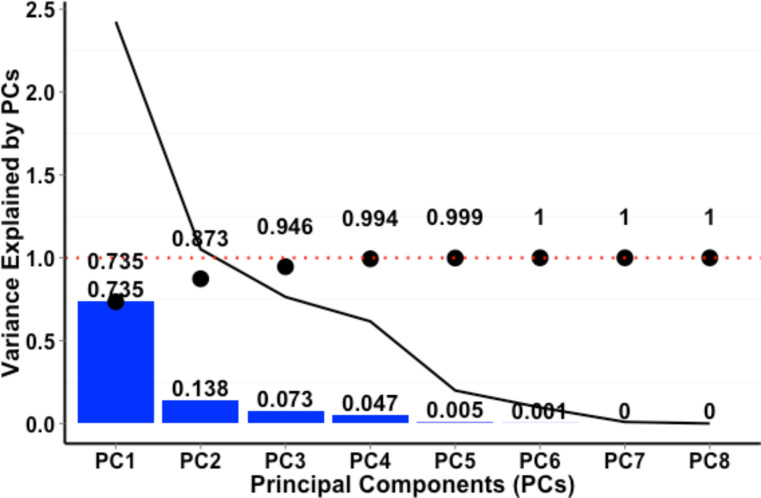
Individual variance for each of the principal components (PCs). Black dots represent the accumulated variance explained by each PC, while the solid line shows the Eigenvalue.

The first two principle components (PCs), whose standard deviations both were greater than 1, explained 87.3% of the total variance of the matrix. As the linear combinations (or weights) of these two PCs were calculated based on all of the input data, they represent all of the particle information. As such, these two PCs were determined to be appropriate to represent the variability in this dataset (Figure 4). These two PCs were selected as the new independent variables, reducing the independent variables’ dimensions from 8 to 2.

**Figure 5:**
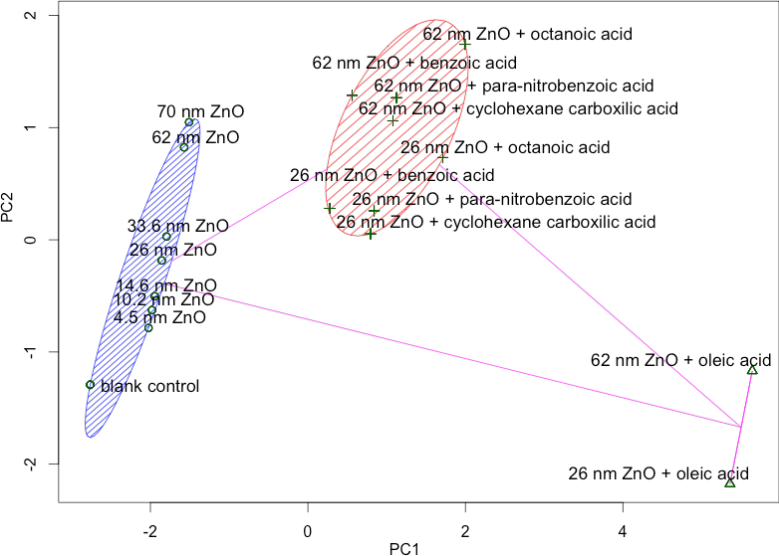
Clustering analysis based on Euclidian distance for ZnO NPs partitioned into 3 clusters. Shown on the left (blue hash marks) are the bare ZnO NPs with the blank control point. In the middle (tan hash marks) are ZnO NPs with 4 different surface chemistries and on the right are the oleic acid modified particles.

Table 3 shows the 8 descriptors all have moderately similar weights in PC1, but Log D, PS and SZ have outstanding weights in PC2. The variable coefficients in the PC1 linear combination all have the same sign, suggesting these parameters have similar effects on the model. In contrast, the sign of the variable coefficients for SZ and PS in PC2 are opposite to the other parameters suggesting these variables help separate the particles. Graphing the PCA scores for PC1 versus PC2 allows for the use of Euclidean distance to identify clusters of similar NPs with respect to their toxicity to embryonic zebrafish. As predicted, the various surface modifications to ZnO NPs resulted in distinct groupings based on these capping agent properties (Figure 5). When partitioned into three clusters, the plot shows a clear separation as: (Group 1) oleic acid; (Group 2) octanoic acid, para-nitrobenzoic acid, cyclohexane carboxylic acid and benzoic acid; (Group 3) bare ZnO with blank control responses (Figure 5). Similar analysis using either four or five clusters shows minor differences compared to the use of three clusters, namely the coated 26 nm NPs (except octanoic acid) separated out of Group 3 in the four cluster calculation and the blank control point separated out of Group 1 in the five clusters calculation in addition to 62 and 70 nm bare ZnO NP separating out of Group 3 (See Supporting Information File 3).

**Table 3:** Rotation of PCA (weighting of each property).

Property	PC1	PC2	PC3	PC4	PC5	PC6	PC7	PC8

SZ^a^	0.188	0.669	0.711	0.072	−0.077	−0.027	0.001	0.000
PS^b^	0.270	0.497	−0.610	0.454	−0.262	0.100	0.063	0.139
SASA^c^	0.404	−0.025	−0.002	0.173	0.844	0.196	−0.090	0.218
RF^d^	0.407	−0.058	−0.063	−0.205	−0.182	−0.320	−0.803	0.062
DE^e^	0.378	−0.001	−0.039	−0.634	−0.222	0.531	0.217	0.274
Log D^f^	0.292	−0.535	0.339	0.538	−0.359	0.142	0.069	0.266
VS^g^	0.410	−0.099	−0.015	0.053	−0.020	0.191	0.063	−0.882
PL^h^	0.408	−0.070	−0.051	−0.150	0.037	−0.714	0.536	0.072

^a^Size; ^b^polar surface; ^c^solvent-accessible surface area; ^d^molar refractivity; ^e^dreiding energy; ^f^distribution coefficient; ^g^van der Waals surface; ^h^polarizability.

### Estimation of toxicity by ordinary kriging method

**Figure 6:**
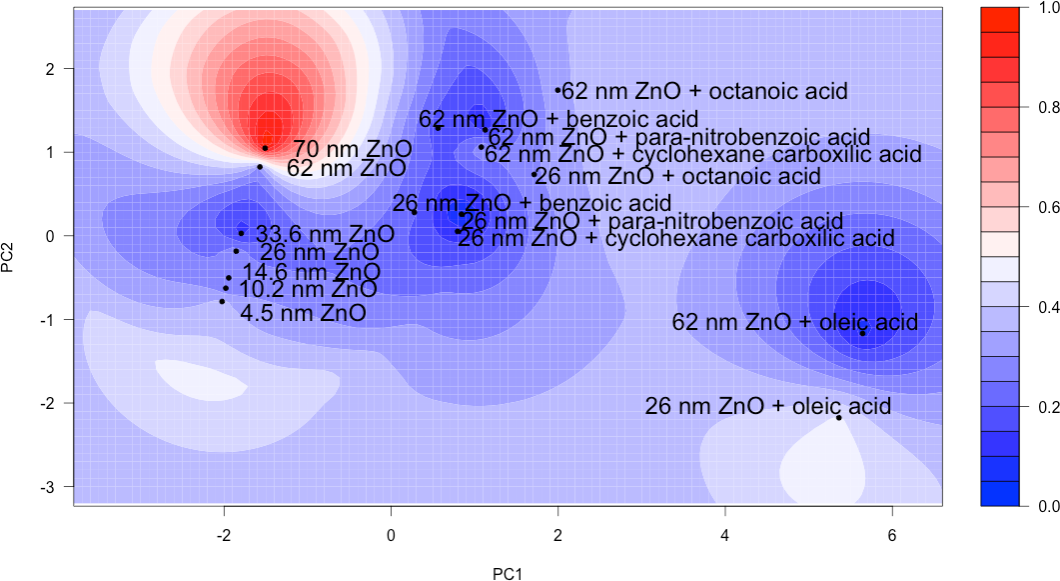
Kriging estimation contour map for embryonic zebrafish exposed to 250 mg/L of each type of zinc oxide nanoparticle using the first two surface chemistry-based principal components as the coordinates and 120 hpf total mortality as response. The coefficient of determination was found to be 0.702.

By using the two most dominant PCs identified earlier as coordinates (XY-direction) and mortality data as the response (Z-direction), we calculated the kriging estimation of mortality. The ordinary kriging method, based on the spherical model, was used to model the mortality of zebrafish embryos at each of the different exposure concentrations for each of the 17 tested NPs. The resulting contour map for the highest exposure concentration (250 mg/L) is shown in Figure 6 and the contour maps for other exposure concentrations can be found in Supporting Information File 4. The coefficient of determination was calculated to determine how well the estimation fit the original data. Similar coefficients of determination were found at each concentration (0.702–0.778).

## Discussion

### ZnO NP toxicity to embryonic zebrafish

Of the numerous sub-lethal endpoints evaluated in our study, most of the significant toxicity resulting from exposure to ZnO NPs was associated with mortality, regardless of the type of surface chemistry found on the nanoparticle. Interestingly, when mortality occurred in the surface functionalized ZnO NPs, it was always within the first 16–18 hours of exposure (observed at the 24 hpf evaluation). Embryos surviving exposure to surface coated ZnO NPs after this initial period had almost 100% survival and no significant developmental abnormalities (see Supporting Information File 2 and Supporting Information File 5). In contrast, the bare ZnO particles resulted in mortality at both 24 and 120 hpf for some materials and a complete lack of toxicity in others. This result supports the hypothesis that outermost surface chemistry is a primary driver of biological interactions, even more than core composition. This finding has been supported in other studies investigating a wide range of NP types [27,41–42].

Given that dissolution and the resulting release of zinc ions and ROS are the primary cause of ZnO NP toxicity [8], it is possible that the lack of late-onset mortality in coated particles is the result of decreased dissolution of these particles [7,21]. It has been reported that the release of zinc ion from ZnO NPs coated with organic molecule can be slower than uncoated ZnO NPs by up to 10 days, due to the protective effect of the surface coating [43]. The idea that coated particles were more benign overall is also supported by the most toxic response being noted for a bare particle (NGZ, Figure 3). In addition, the observed mortality at 24 hpf for some of the surface functionalized particles could have been due to either residual impurities or zinc ions, as any dissolved zinc would have remained in the exposure media due to the static nature of these experiments. The delayed mortality response in the bare ZnO particles could also relate to the onset of mouth-gaping behavior during fish development that led to increased uptake over the exposure period; however, this would likely have occurred with the coated particles as well unless this was specific to zinc ion uptake or direct impacts of generated ROS.

Only one ZnO NP (NGZ) caused any significant sublethal impacts in the developing fish with notochord malformations as well as significant malformations of the swim bladder. Despite NGZ being an uncoated ZnO NP, its unique toxicity relative to the other non-coated ZnO NPs suggests some other features, such as crystal morphology, may be contributing to the observed differential toxicity. It is known that ZnO NPs with sharper angles have been noted to contribute to lower viability in cell culture studies with A549 and HT29 cells [30]. Similar morphology effects on toxicity have been observed in studies of manganese oxide, where the sharp points and edges were found to generate more ROS than smooth surfaces [44]. We tested this hypothesis by comparing X-ray diffraction (XRD) results for NGZ relative to a representative sample of the other bare ZnO NPs (Sigma-Aldrich, 63 nm, NBI_0215) using a Bruker-AXS D8 Discover XRD instrument (Karlsruhe, Germany and Madison, WI). No differences in the lattice parameters were identified, thus other intrinsic factors must be contributing to the unique toxicity of this commercial ZnO NP (see Supporting Information File 6).

Since the size of the ZnO NP did not elicit any general trends in the toxic responses observed, it is likely that surface features of the particle impacting interactions with biological membranes may drive toxicity more than the size of the particle itself. NP agglomeration in aquatic environments often occurs and can be influenced by physicochemical properties of the particle surface and environmental factors affecting the zeta potential [27,45–46]. Therefore, it is possible that the agglomeration of the particles in the fishwater media could indirectly affect dissolution or interactions with the developing embryo. Previous studies have found that uncoated ZnO NPs form smaller aggregates on the surface of bacteria than are formed in suspension [47], and this type of surface aggregation cannot be ruled out as a contributing factor in our results. Previous studies with the freshwater crustacean *Daphnia magna* based on 30, 80–100 and 200 nm ZnO NPs found that toxicity was not dependent on the primary particle size [11]. This is similar to what we found for the bare ZnO NPs in our study which range from 4 to 70 nm.

Overall, the toxicity results suggest that surface features do impact ZnO NP toxicity. In addition, the evaluation or mortality at multiple time points during development is useful in modeling nanoparticle–biological interactions using zebrafish [45].

### PCA

PCA combines as much information as possible to provide an overview of the known and unknown relationships between inherent NP features and developmental toxicity. The eight original intrinsic properties descriptors were correlated with each other based on similarities in value of PC1 weights, however more separation was gained using the weighting of PC2 (Table 3). The latent factor suggested by PC2 is the Log D, which plays a different role in the ZnO NPs toxicity compare to size and polar surface effects. The unique clustering of both sizes of oleic acid functionalized particles suggests the properties of this ligand are somewhat unique relative to the others, perhaps due to the long chain length (Figure 2) and high hydrophobicity of oleic acid (Table 2). Oleic acid coated ZnO NPs which have the highest hydrophobicity (Log D 5.62), showed the smaller size one was more toxic and separated from the remainder of the coated particles in the PCA. In contrast, the remaining surface functionalized particles all had much lower log D values (Table 2) and clustered together in our analysis. The Log D calculations can be affected by electrolyte concentration, however in our study this was too small (Cl^−^ 0.0174 mol/L and Na^+^, K^+^ 0.0165 mol/L) to affect its value relative to water, thus these inherent properties value are expected to reflect the true properties in fishwater. This suggests that future studies should continue to investigate surface features impacting the hydrophobicity of the particle as potential contributors to toxicity. However, this result depends on our assumption that the coating chemicals dominate the hydrophobicity of the metal oxide NP [22]. Even when surface chemistry is constant among ZnO NPs, differential particle morphology and variations in the suspension media will likely affect dissolution and alter the hydrophobicity in comparison to theoretical values of Log D [30].

Other intrinsic properties not considered, such as the proportional amount of ligand coverage on the surface of the nanoparticle, may improve model performance further. Unfortunately this level of detailed characterization of the surface chemistry is often unavailable from manufacturers and is cost- and time-intensive to determine for a wide range of surface chemistries. Further refinement of the model could likely also be achieved by including more complex calculation of intrinsic values that are based on the actual ligand-nanoparticle structure rather than surface ligand structure alone (in the absence of consideration of bonding with the NP). In studies of multiple engineered nanoparticles, it is nearly impossible to set single variable control groups due to correlated descriptors and constraints in characterizing NPs in the experiment conditions. However, we have shown that PCA can be used as a valuable alternative method to estimate the relative effects of multiple inherent properties simultaneously to support the development of predictive models that will allow for the development of safer ZnO materials.

Based on the large differences in molecular properties between the organic surface coatings and the bare zinc oxide properties (Table 2), it was expected that each group would separate during clustering analysis, as was the case with this data (Figure 5). Identified clusters suggest that a set of appropriate intrinsic properties of surface chemistry can be used to partition NPs into different groups. The 17 ZnO NPs partitioned into clusters that were fairly easy to identify using only capping agent properties. However, with more complex surface structures, overlap between clusters might happen making determination of the cluster number the first concern. Although there are several algorithms to decide the cluster number, the lack of robust data sets such as this preclude a current understanding of which algorithm may be appropriate [48].

### Kriging estimation

Based on the two most dominant PCs that explained 87.3% of the variance in the toxicity data, we performed the kriging estimation at each of the exposure concentrations. Interestingly, the exposure concentrations had little influence on the coefficients of determination with similar values being determined at each concentration (Figure 6, Supporting Information File 4). Kriging estimation further elucidated the impacts of NP size. Based on Figure 6, we can see that the largest bare particle (NGZ) also has the highest mortality (Figure 3B) and the cluster 2 surface modified 26 nm particles were predicted to have overall lower toxicity than the larger versions of the same particle. However, this trend does not hold for the oleic acid functionalized particles as the smaller particles are predicted to be higher in toxicity. Therefore, outermost surface chemistry continues to play a more important role in determining toxicity.

## Conclusion

The observed toxic responses of developing zebrafish embryos to ZnO NP exposure varied with surface chemical modification and were only minimally impacted by particle size. Only NGZ, a bare ZnO NP, had relatively high toxicity, suggesting specific product features of bare ZnO NPs drive toxicity. This work has shown that large databases of similar NPs with varying surface features studied under identical experimental design protocols, are invaluable in the development of models of nanoparticle-biological interactions. We have shown that intrinsic features of NPs, particularly those encompassing the outermost surface chemistry, are useful in the classification and clustering of NP toxicity data. Our finding that hydrophobicity was the strongest determinant of toxicity of the many surface features we investigated will contribute to the development of predictive models of ZnO NP-biological interactions. We have found that PCA is a useful tool for reducing numerous surface molecular properties to fewer dimensions. Future development of highly accurate predictive models will depend on detailed information provided by in silico modeling and analysis of the outermost surface of the nanoparticle. Overall, identification of specific material features, such as outermost surface chemistry, that drive biological interactions appears feasible and models such as this should continue to be tested and refined to achieve safer design principles for the manufacture of ZnO NPs.

## Experimental

### Nanomaterials

The ZnO NPs with different capping agents and sizes were obtained from a variety of commercial and research laboratories (Table 1). More detailed characterization of the nanomaterials are also available on the open-source Nanomaterial-Biological Interactions Knowledgebase [39] provided by Oregon State University.

### Estimation of surface chemical parameters

The eight surface chemical descriptors we utilized were size, hydrophobicity (Log D), polarizability, polar surface area, van der Waals surface area, solvent accessible surface area, molar refractivity and Dreiding energy (Table 2). Except for the primary particle sizes (which were provided by manufacturers), the seven other intrinsic properties of capping agents were calculated by software (Table 2). Log D is calculated using Advanced Chemistry Development (ACD/Labs) Software version 11.02. PL is retrieved from ChemSpider (Mar. 2014), which was predicted by ACD/Labs Percepta Platform - PhysChem Module. VDW surface (VS), PS, SASA, RF and DE were calculated in Marvin Beans (version 6.2.2, Cambridge, MA). All inherent chemical properties were calculated based on the pH used in zebrafish toxicity test.

### Embryonic zebrafish assay

Wild-type 5D zebrafish (*Danio rerio*) embryos were obtained from group spawns of adult fish housed at the Sinnhuber Aquatic Research Laboratory at Oregon State University (Corvallis, OR). All NP dilutions and exposures were conducted in fish water (FW). The FW was prepared with 0.26 g/L Instant Ocean salts (Aquatic Ecosystem, Apopka, FL) combined with approximately 0.01g NaHCO_3_ pH buffer in reverse osmosis water (pH 7.0–7.4, conductivity 450–600 μS). Embryos were collected at 6 hours post-fertilization (hpf) and maintained at 27 °C under 14/10 light and dark cycle. Embryos were exposed individually in 96-well plates to 7 different concentrations (0.016 to 250 mg/L) of each type of ZnO NP suspended in FW. Prior to exposure, embryos were dechorionated at 6 hours post-fertilization (hpf) with pronase (Sigma-Aldrich) and then rinsed several times with FW [25]. The control groups are FW alone without NPs present. A total of 21 endpoints were observed during development at 24 and 120 hpf that included mortality as well as morphological, behavioral and developmental endpoints in sub-lethal exposures [49]. The 19 sub-lethal endpoints include developmental progression (DP), spontaneous movement (SP), notochord (N), yolk sac edema (Y), axis (A), eye (E), snout (Sn), jaw (J), otic (O), heart (H), brain (B), somite (So), pectoral fin (PF), caudal fin (CF), pigment (P), circulation (C), trunk (T), swim bladder (SB), and touch response (TR).

### Statistical analysis

Due to the non-parametric nature of the data and the small sample size (<30 embryos for each exposure concentration), the Fisher’s exact test (Sigma Plot v12.0, San Jose, CA) was used to analyze individual endpoints recorded at 24 and 120 hpf [50]. *P*-value was calculated based on two-tailed test and a *p* ≤ 0.05 significance level was maintained for all analyses. Mortality data was compared between NPs with the same capping agent but different sizes using two-way analysis of variance (R, version 3.1.0, Vienna, Austria).

Principal component analysis (PCA) was conducted in R using the primary particle size and seven intrinsic properties of NPs’ surface chemistry shown in Table 1 and Table 2, respectively. To include control groups (blank group) in the analysis, all of the intrinsic NP properties are set to 0 for the blank groups. The same intrinsic properties were used for all exposure concentrations (0.016 mg/L to 250 mg/L) for a given particle type. The normalization process was conducted on the dataset as a matrix in PCA, with the mean of normalized data equal to 0 and standard deviation equal to 1. Then 8 different linear combinations consisting of 8 independent variables and their coefficients (also called “rotation” in R) were generated as new vectors, called principal components (PCs). The converted value, called score (stored as “x” in R), was used to model the toxic responses. The ordinary kriging was conducted in R using the additional “Kriging” and “gstat” packages.

## Supporting Information

File 1 Zebrafish malformation and behavioral data. The 19 sub-lethal endpoints are developmental progression (DP), spontaneous movement (SP), notochord (N), yolk sac edema (Y), axis (A), eye (E), snout (Sn), jaw (J), otic (O), heart (H), brain (B), somite (So), pectoral fin (PF), caudal fin (CF), pigment (P), circulation (C), trunk (T), swim bladder (SB), and touch response (TR).

File 2 Fisher’s exact test *p*-value. The 19 sub-lethal endpoints are developmental progression (DP), spontaneous movement (SP), notochord (N), yolk sac edema (Y), axis (A), eye (E), snout (Sn), jaw (J), otic (O), heart (H), brain (B), somite (So), pectoral fin (PF), caudal fin (CF), pigment (P), circulation (C), trunk (T), swim bladder (SB), and touch response (TR). Included are three mortality (M) endpoints at 24 and 120 hours post fertilization after the exposure to ZnO NP and the sum of two M.

File 3 Cluster analysis of converted data using Euclidean distance to partition into A) 3, B) 4, C) 5, D) 6 clusters.

File 4 Kriging estimations of zebrafish mortality data at A) 0.016 ppm, B) 0.08 ppm, C) 0.4 ppm, D) 2 ppm, E) 10 ppm, F) 50 ppm.

File 5 Embryonic zebrafish mortality at 24 and 120 hours post fertilization after ZnO NP exposure.

File 6 XRD analysis of three different ZnO NPs.
