# Supplementary figures and images for: Influence of surface chemical properties on the toxicity of engineered zinc oxide nanoparticles to embryonic zebrafish
Source: Beilstein J Nanotechnol. 2015 Jul 20;6:1568–79. doi: 10.3762/bjnano.6.160 (PMC4578392; doi:10.3762/bjnano.6.160)

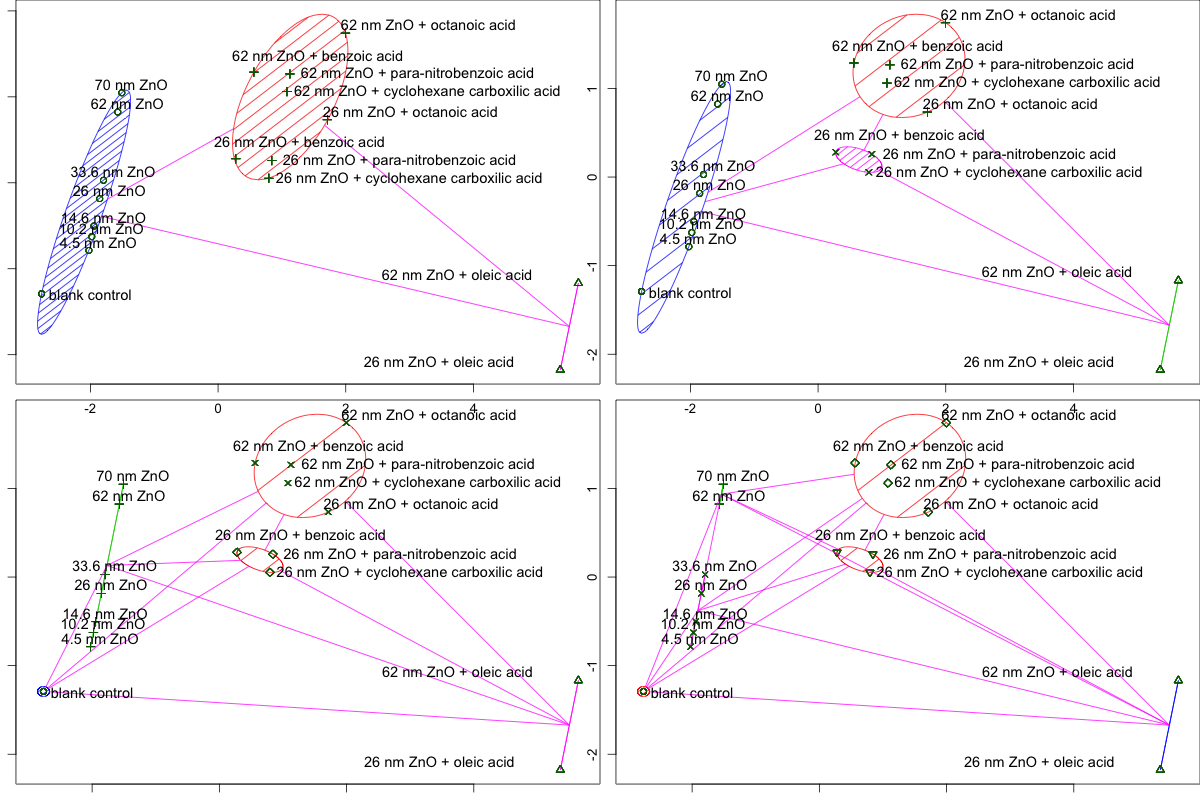

Supplement: File 3 — Cluster analysis of converted data using Euclidean distance to partition into A) 3, B) 4, C) 5, D) 6 clusters. [file Beilstein_J_Nanotechnol-06-1568-s003.png]

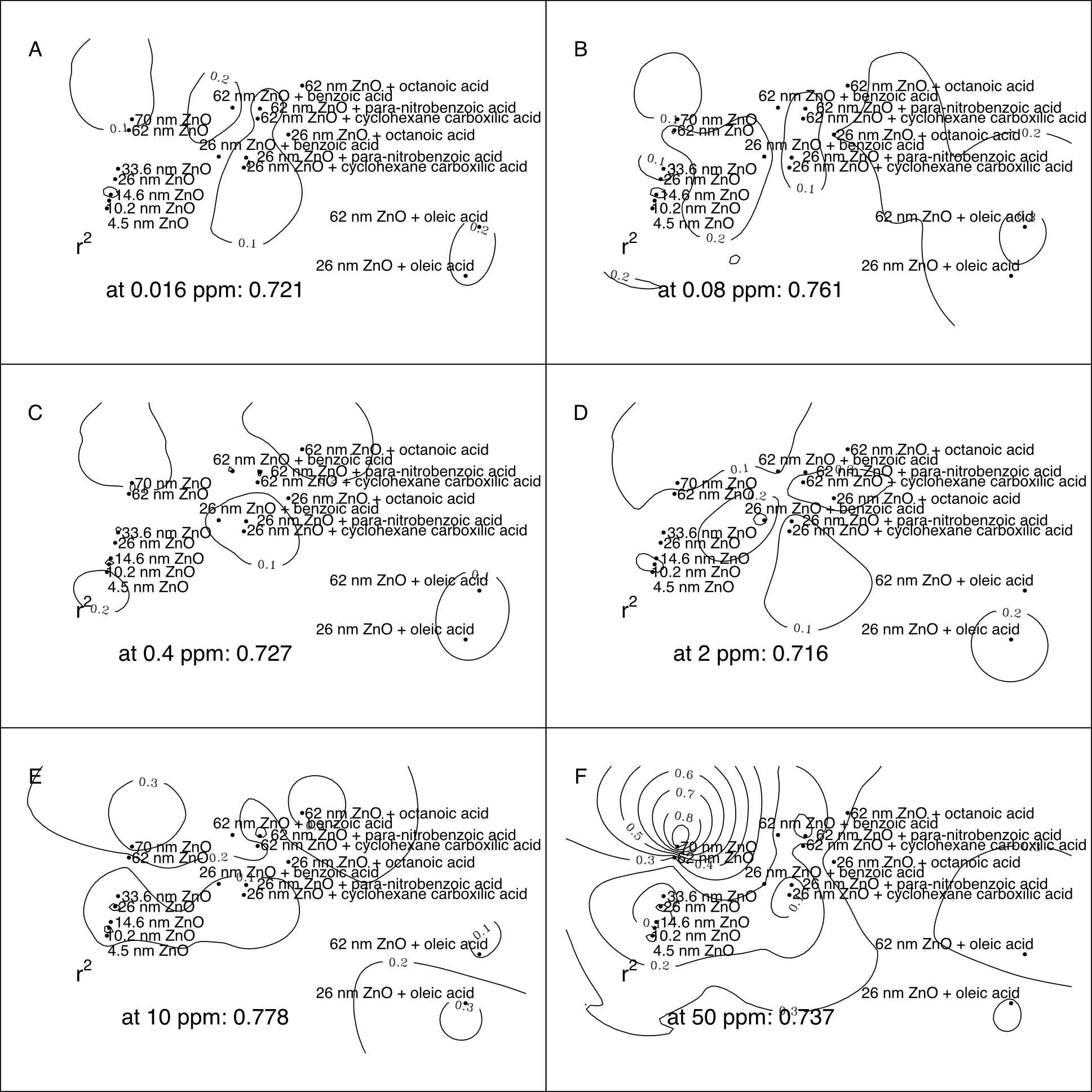

Supplement: File 4 — Kriging estimations of zebrafish mortality data at A) 0.016 ppm, B) 0.08 ppm, C) 0.4 ppm, D) 2 ppm, E) 10 ppm, F) 50 ppm. [file Beilstein_J_Nanotechnol-06-1568-s004.png]

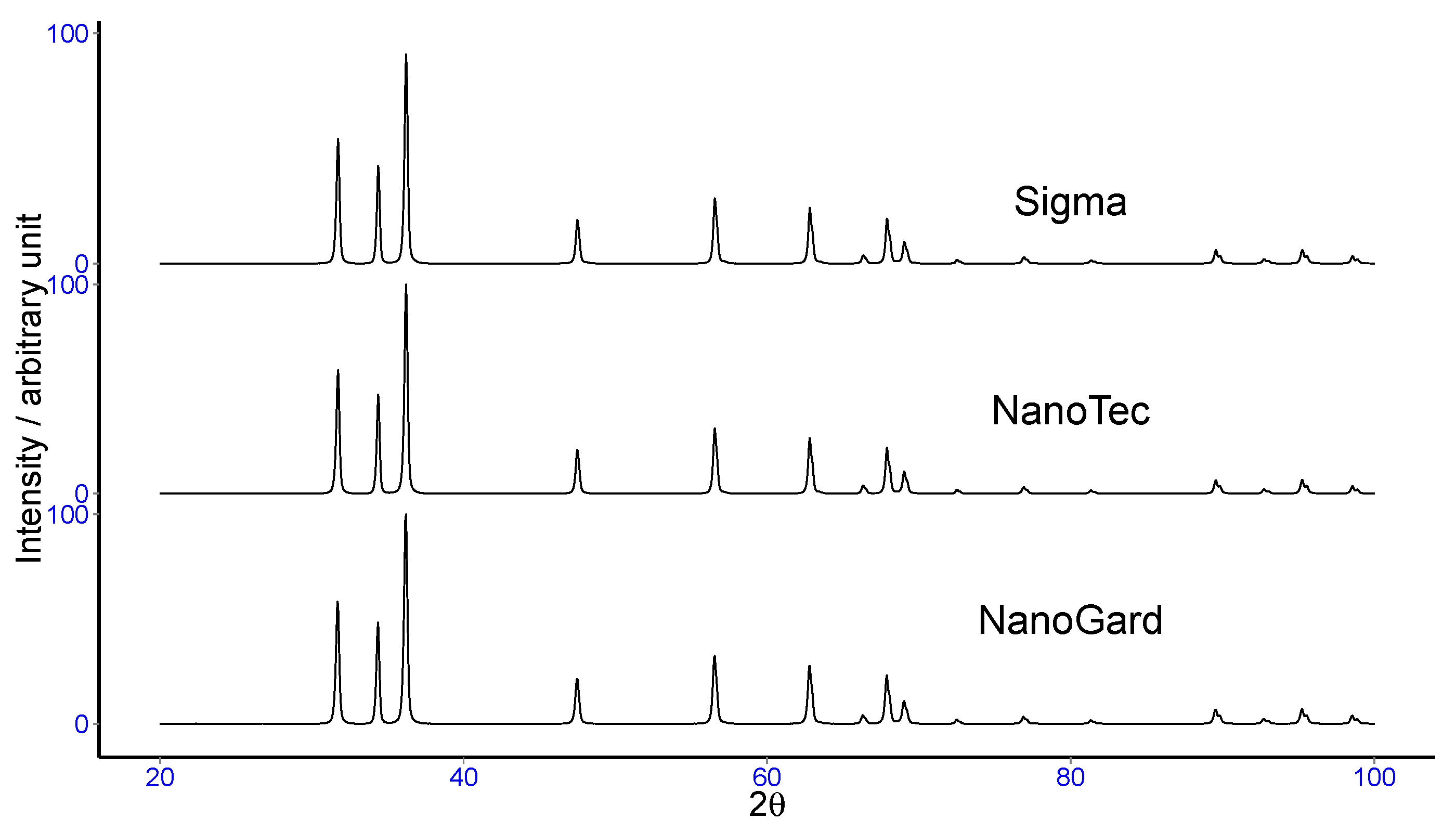

Supplement: File 6 — XRD analysis of three different ZnO NPs. [file Beilstein_J_Nanotechnol-06-1568-s006.png]
